# Disguising Reddit sources and the efficacy of ethical research

**DOI:** 10.1007/s10676-022-09663-w

**Published:** 2022-09-10

**Authors:** Joseph Reagle

**Affiliations:** Communication Studies, Northeastern University, 215 Holmes Hall, 43 Leon St, 02115 Boston, MA USA

**Keywords:** Ethics, Research, Online, Reddit, Disguise, Fabrication

## Abstract

Concerned researchers of online forums might implement what Bruckman (2002) referred to as *disguise*. Heavy disguise, for example, elides usernames and rewords quoted prose so that sources are difficult to locate via search engines. This can protect users (who might be members of vulnerable populations, including minors) from additional harms (such as harassment or additional identification). But does disguise work? I analyze 22 Reddit research reports: 3 of light disguise, using verbatim quotes, and 19 of heavier disguise, using reworded phrases. I test if their sources can be located via three different search services (i.e., Reddit, Google, and RedditSearch). I also interview 10 of the reports’ authors about their sourcing practices, influences, and experiences. Disguising sources is effective only if done and tested rigorously; I was able to locate all of the verbatim sources (3/3) and many of the reworded sources (11/19). There is a lack of understanding, among users and researchers, about how online messages can be located, especially after deletion. Researchers should conduct similar site-specific investigations and develop practical guidelines and tools for improving the ethical use of online sources.

## Introduction

Reddit is known as the “front page of the web,” claiming “52 M + daily active users” and “100K + communities” (Reddit, 2021﻿). Millions of Redditors, including minors and other vulnerable populations, have thousands of subreddits to discuss extraordinarily specific and sometimes sensitive topics, including sexuality, health, violence, and drug use.

Given the public prominence, breadth, and depth of Reddit’s content, researchers use it as a data source. Proferes et al., (2021) identified 727 such studies published between 2010 and 2020-May. They found that only 2.5% of their studies claimed to paraphrase compared to the 28.5% of the studies that used exact quotes. Researchers who do paraphrase write of limiting the locatability of sources and possible consequent harm. (The studied reports are not quoted or cited directly, see the “Ethics” section below.) I am not aware of cases, fortunately, of online users coming to harm because of information in research reports. That doesn’t mean it hasn’t happened, and given the sensitivity of topics and vulnerability of sources, additional scrutiny could be consequential to users’ health, relationships, employment, and legal standing. (For a more general discussion of potential harm, sensitive topics, and sources, see Franzke et al., (2020), § 3.2.5.) Additionally, I note that users need not be personally identified to feel embarrassed, to be harassed, or to be forced to abandon a long-held pseudonym. And researchers themselves, whose use of public sources is thought to be outside of human subjects review, might face embarrassment or repercussions nonetheless if a source complains. Paraphrasing sources’ prose might mitigate such outcomes.

Verbatim quoting and paraphrasing are two practices within a spectrum of what Bruckman (2002) identified as *disguise*, which can range from none to heavy. Disguise can also include altering usernames and the context of a message, such as the time and forum of posting.

I analyze 22 Reddit research reports: 3 of light disguise, using verbatim quotes, and 19 of heavier disguise, claiming to reword phrases. I test if their sources can be located via three different search indexes (i.e., Google, Reddit, and RedditSearch). I was able to locate all of the verbatim sources (3/3) and many of the reworded sources (11/19). I also interview 10 of the reports’ authors about their sourcing practices, influences, and experiences. These conversations reveal that there is a lack of coherent practice and guidance on effective disguise, the importance of search and archival affordances, and how errors can arise amidst multi-author collaborations and during the review and revision process. Most importantly, these interviews identify exemplary practices, such as researchers testing their own disguises.

The present work does not address whether researchers *should* disguise their sources. This decision depends on the type of research, the sensitivity of the topic, the vulnerability of the sources, and the attributes of the venue. Rather, my concern is empirical: when researchers choose to employ disguise, does it work? And what, then, can we do to improve the practice?

## Background and terminology

### Reddit and sensitive topics

Reddit was founded in June 2005 as a pseudonymous-friendly site for users to share and vote for links they had read (i.e., “I read it.”) Reddit’s development as a forum of forums, where users could trivially create subreddits, each with its. own moderators, led the website to succeed over its link-sharing peers.

Like Twitter and Wikipedia, Reddit serves an extraordinary corpus of mostly public data. That is, while there are private and quarantined subreddits, the vast majority of content is *public*: transparently accessible to any web browser or search engine. More so than Wikipedia and much of Twitter, Reddit hosts discussions of a personal character. Subreddits on sexuality, health (including mental health and eating disorders), interpersonal abuse and violence, and drug use and cessation have been topics of research. Reddit is a compelling and accessible venue, but with sensitive – even if public – information.

### Ethics and Online Research

The practice of online research has been accompanied by discussion of how to do so ethically (Eysenbach & Till, 2001; Flicker et al., 2004; Mann & Stuart, 2000; Reid, 1996; Smith & Murray, 2001; Waskul & Douglas, 1996). And the issues noted by Siang (1999) over two decades ago remain salient today: of “the blurred distinction between public and private domains,” “the ease of anonymity or pseudonymity,” “the suspension of temporal barriers with the recording and archiving of communications,” and “the relatively low cost, easy to use, technological means to facilitate tracking of participants.”

An intuitive approach to these early concerns was to apply existing research guidelines to the online context, such as those from the American Psychological Association (King 1996) and other disciplinary and national societies. At the same time, the Association of Internet Researchers (AoIR) was constituted as a transdisciplinary endeavor, which created and maintains guidelines for online research (Ess & Committee 2002; Franzke et al., 2020).

Institutional review boards (IRBs) can also be a source of guidance and review. Like much of the disciplinary guidelines, however, their focus tends to be on human subjects research, where there is an interaction between researchers and subjects. Most Reddit research (86.1%) makes no mention of “IRB” or “ethics review.” Of those that do, the majority (77.2%) note an exempt status, though it’s unclear if this is “an official designation given by a review board or whether the authors made this judgment themselves” (Proferes et al., 2021, p. 14).

What is clear is that there is no widely accepted and consistent practice when it comes to reporting excerpts of public content. Systemic literature reviews show this (Ayers et al., 2018; Proferes et al., 2021), as will the present work. For those concerned with disguising public data, there’s little guidance on how to do so effectively.

### Online Sites, subjects, and sources

The incoherent approach to public data is related to a lack of agreement about terminology and substance. What should researchers call those whom they research online? I distinguish between *subjects*, those with whom researchers interact, and *sources*, authors of public content with whom researchers do not interact. (Recall that I use public to mean content that is transparently accessible to any web browser or search engine.) There is also the question of Reddit terminology. Following the architecture of Reddit, I distinguish between *posts* and their subsequent *comments* within a *thread*. I refer to posts and comments, generically, as *messages*.

Beyond terminology, what should researchers do? There is substantive disagreement compounded by different understandings of terms. Sharf (1999, p. 253), for example, argued that researchers should contact public sources “in order to seek consent” and “implied consent should not be presumed if the writer does not respond.” Rodham & Gavin (2006) responded “that this is an unnecessarily extreme position to take” and wrote, “messages which are posted on such open forums are public acts, deliberately intended for public consumption.” Presently, I analyze published research reports and seek their sources, without consent. Unlike Sharf’s study of a breast cancer email list (“public” because the list is “open” for anyone to join), published reports are closer to Rodham and Gavin’s sense of the term (i.e., “intended for public consumption”).

Increasingly, researchers are engaging in site-specific considerations, which requires contextual ethical reasoning, be it at Wikipedia (Pentzold 2017), at sites where “we are studying people who deserve credit for their work” (Bruckman et al., 2015), or public sites where people, nonetheless, discuss sensitive topics or share images (Andalibi et al., 2017; Ayers et al., 2018; Chen et al., 2021; Dym & Fiesler, 2020; Haimson et al., 2016). For example, on Twitter, Fiesler & Proferes (2018) found that “few users were previously aware that their public tweets could be used by researchers, and the majority felt that researchers should not be able to use tweets without consent. However, we find that these attitudes are highly contextual, depending on factors such as how the research is conducted or disseminated, who is conducting it, and what the study is about.” Additionally, as I will show, specific websites have affordances that affect how sources can be located (e.g., novel search capabilities or external archives).

### De-Identifying, Anonymizing, fabricating, and Disguising

Researchers who attempt disguise note that their sources might be struggling with health, sexuality, or drug use, and additional scrutiny might cause them harm. For the reasons that follow, I speak of *disguising* public sources to prevent them from being *located*.

Bruckman (2002) identified a spectrum of disguise, from none to heavy. Under light disguise, for example, “an outsider could probably figure out who is who with a little investigation.” The forum is named, usernames and other details are changed, yet “verbatim quotes may be used, even if they could be used to identify an individual.” Under *heavy disguise*, some false details are introduced and verbatim quotes are avoided if a “search mechanism could link those quotes to the person in question.” If the heavy disguise is successful, “someone deliberately seeking to find a subject’s identity would likely be unable to do so.” *Moderate disguise* is “a compromise position … incorporating some features of light disguise and some of heavy disguise, as appropriate to the situation.” Kozinets (2015, p. 3473) adopted this notion in his discussion of ethical netnography though he used the term *cloaking* “to emphasize the researcher’s protective actions rather than the state of the participant.” This is a good point, but *disguise* is the more common term in the literature.

In commercial contexts, enterprises use *sanitization* to remove sensitive information such as “credit card numbers, email addresses and Social Security Number (SSN)” (Nguyen & Cavallari 2020, pp. 37–38). In human subjects research, such as healthcare, *de-identification* “involves the removal of personally identifying information in order to protect personal privacy” (Guidelines for Data de-Identification or Anonymization, 2015). *Anonymized* is sometimes used synonymously with de-identified, or can have a stronger connotation of data being rendered incapable of being re-identified. I avoid *anonymized* because it is far too an assured word given the known cases of failure (Ohm 2010). And in public data contexts, there might not be personally identifiable information to speak of given the use of pseudonyms. Even so, users need not be personally identified to feel exposed or embarrassed, to be harassed, or to be forced to abandon a long-held pseudonym.

Introducing false or combined details about a source has been referred to as *fabrication*, a tactic of heavy disguise. The practice is not limited to prose and can include visual content, such as a profile picture in a screenshot (Haimson et al., 2016). This practice can conflict with traditional notions of research rigor and integrity. Markham (2012) argues that if done with care, fabrication can be the most ethical approach. If not done with care, however, fabrication can lead to suspicions of fraud (Singal, 2016).

*UnGoogling* has been used for “obscuring published data and analysis from index, search, and retrieval for ethical purposes” (Shklovski & Vertesi, 2013, p. 2172). And *obfuscating* has been used to speak of the “deliberate addition of ambiguous, confusing, or misleading information to interfere with surveillance and data collection” (Brunton & Nissenbaum 2015, p. 1). *UnGoogling* is too service-specific and is more often used to describe users removing themselves from the Google ecosystem, such as abandoning Android for iOS. *Obfuscation*’s most common use is to describe users protecting their privacy rather than as a research practice.

I examine research reports who *disguise* their public sources to keep them from being *located*.

### Locating sources

What of the substance, the process, of locating research subjects or sources? Sometimes the ethnographic subjects themselves, of a small town, for example, can recognize themselves and their neighbors. Sometimes real-world events, such as the occurrence of a murder, provide a clue to the public (Reyes, 2017, n. 9; Singal, 2015). And when a researcher from a top-tier New England university describes their research using undergrads from a top-tier New England university, the subjects are probably their students. Online, messages’ prose style (Narayanan et al., 2012), timing (Johansson et al., 2015), and network relationships (Zhou et al., 2016) serve as digital fingerprints (Brown & Abramson, 2015), amendable to digital forensics (Guarino, 2013), which can lead to online accounts and even personal identities being linked together (Backes et al., 2016). For example, Narayanan & Shmatikov (2009) were able to re-identify a third of users in their “anonymous” Twitter graph who also had a Flickr account “with only a 12% error rate … even when the overlap between the target network and the adversary’s auxiliary information is small.”

As far back as the 1990s, King (1996) faulted Finn & Lavitt (1994) for disguising sources’ names, but not that of the sexual abuse forum or the date and time of posts. More recently, Zimmer (2010) critiqued researchers from a top-tier New England university for creating a “Tastes, Ties, and Time” Facebook dataset that was improperly – perhaps impossibly – “anonymized.” The data was obtained by Harvard Resident Advisors acting as research assistants and scraping their Facebook friends lists. And once the school and cohort were known, other aspects of students’ tastes, ties, and activity made re-identification possible (e.g., being the only student from a specific country in the dataset). Journalists, too, sometimes participate. At the *New York Times*, Barbaro & Zeller (2006) reported on – and confirmed – the potential to locate sources in an AOL dataset. A decade later, in the same newspaper, Singer (2015), wanting to speak to a source in a research study, was able to identify, contact, and interview the subject.

Concerned researchers have started to assess how often usernames, quotations, and media are included in research reports. Ayers et al., (2018) analyzed 112 health-related papers discussing Twitter and found 72% quoted a tweet, “of these, we identified at least one quoted account holder, representing 84%.” When usernames were disclosed, in 21% of the papers, all were trivially located. Ayers et al. wrote that these practices violate International Committee of Medical Journal Editors (ICMJE) ethics standards because (1) Twitter users might protect or delete messages after collection, and (2) revealing this information has no scientific value.

Proferes et al., (2021) performed a systematic overview of 727 research studies that used Reddit data and were published between 2010 and 2020-May. They found “Sixty eight manuscripts (9.4%) explicitly mentioned identifiable Reddit usernames in their paper and 659 (90.7%) did not. Two hundred and seven papers (28.5%) used direct quotes from users as part of their publications, 18 papers used paraphrased quotes, noting they were paraphrased (2.5%) and 502 (69.1%) did not include direct quotes” (Proferes et al., 2021, p. 14).

I make no claim as to whether sources should be disguised. Rather, I ask if a researcher chooses disguise, does it work? *Can the original message used by a researcher be located?* If so, the full message, associated username, and context (i.e., subreddit, thread, and posting history) are then available. This, itself, could be revealing or linked with other information, including personally-identifying information.

## Method

I collected two sets of research reports. In 2020, I sought ethnographic-type research reports since 2015 that included Reddit messages. I searched via Google using keywords such as “AoIR guidelines,” “privacy,” “verbatim,” and “fabrication.” I found three reports using light-disguise with verbatim phrases and three claiming heavier disguise with reworded phrases. In 2021, as part of a panel proposal, I discussed this work with two of the authors of a systematic review of Reddit (Proferes et al., 2021), and they kindly shared their list of reports that “paraphrased” Reddit messages, adding 16 new reports to my initial set. Because *paraphrase* can connote significant change, I use the term *reword*, which can be as minimal as inserting an adjective or altering a place or name. The final corpus, then, included 22 reports, with 19 claiming to reword.

From each report, I collected phrases of more than ten words because any less than that is too short for meaningful searches. I excluded phrases from subreddit documentation such as sidebars, wikis, or FAQs; these have multiple authors and are informative rather than personal disclosures.

The process of locating Redditors’ original messages was idiosyncratic: intensive, manual, and subjective. I performed exact searches (using quotation marks) and inexact searches across the whole phrase and fragments of novel-seeming prose. Near the end of this work, and hoping to share a method of scrambling phrases and testing disguises, I wrote a script that automated the invocation and opening of search query results (Reagle & Gaur 2022). Even so, I had to use discretion with how many search results to review, usually no more than the first page or 20 results – each search service returns results differently. I made no effort to personally identify Reddit users. However, locating sources, as I attempted, could be the first step in the distinct process of identifying users.

After my initial analysis, I emailed the research reports’ authors and asked if they would speak with me. If so, and they completed the consent form, I began with five questions about their practice, rationale, influences, and thoughts about my efforts. We worked to identify weaknesses to avoid and strengths to emulate as part of research and publication. One interview was a ~ 30-minute voice communication, others were via email exchanges with each subject. Interviewees were allowed to review my characterization of their work and our discussion in this report.

Though I used public research reports and their own Reddit sources in my analysis, they are not identified, cited, or quoted. I wanted candid interviews with researchers free of possible embarrassment. I hope that “someone deliberately seeking to find a [subject’s or source’s] identity would likely be unable to do so” (Bruckman 2002). That said, other Reddit researchers who are conversant with the literature could make guesses about the identity of research sources. Should this happen, I believe my sources have plausible deniability.

This method was specified as part of Institutional Review Board application #20-08-30 and “approved” as DHHS Review Category #2: “Exempt… No further action or IRB oversight is required as long as the project remains the same.”

## Analysis and discussion

Table 1 describes the reports’ approaches to phrases, number of sources, and how many were located. The rightmost column has strengths (bold Ⓢ) to emulate and weaknesses (Ⓦ) to avoid in creating effective disguise relative to reports’ stated policy, actual practice, and ease of location. Importantly, all reports articulated a policy of disguise in their approach to sources, even if weak (i.e., removed usernames but included verbatim quotes).

**Table 1:** Research reports and results (“i” = interview)

report	approach	sources	located	strength or weakness
V1	verbatim	18	17	Ⓦ included sources contrary to policy
V2	verbatim	17	15	Ⓦ overconfident about identifying pseudonyms
V3 _i_	verbatim	6	6	Ⓦ inconsistencies arising from revise & resubmit
R1	reworded	2	0	Ⓢ **relied on interviews**
R2 _i_	reworded	5	5	Ⓦ disclosed subreddit and thread
R3 _i_	reworded	8	0	Ⓢ **tested rewording**
R4 _i_	reworded	1	0	Ⓢ **10 years over 3 subreddits**
R5	reworded	3	1	Ⓦ failed to reword
R6	reworded	8	3	Ⓦ inserted 1 adjective in 20 word phrase
R7	reworded	11	1	Ⓦ preserved novel words, which are easily located
R8	reworded	13	0	Ⓢ **12 + subreddits**
R9 _i_	reworded	11	0	Ⓢ **12 + subreddits**
R10	reworded	9	4	Ⓦ 3 verbatim + 1 simple contraction
R11	reworded	2	0	Ⓢ **unspecified subreddits**
R12 _i_	reworded	7+	7+	Ⓦ multi-author process yielded weak-to-no disguise
R13	reworded	4	0	Ⓢ **significant rephrasing**
R14 _i_	reworded	17	12	Ⓦ specified year
R15 _i_	reworded	4	4	Ⓦ single year, single subreddit
R16 _i_	reworded	11	7	Ⓦ only change names/places; multiple sources per thread
R17	reworded	3	0	Ⓢ **dozens of subreddits**
R18 _i_	reworded	3	3	Ⓦ specified subreddits; large excerpts
R19	reworded	13	6	Ⓦ only slight punctuation changes

### Searching reddit and the meaning of deletion

Authors V1 and V2 both relied on the fact that Redditors are typically pseudonymous. They included verbatim quotes *without* the authors’ usernames (i.e., light disguise).

V2 claimed that because pseudonyms are encouraged, the quoted Redditors could not be traced. This claim is *highly* probable, but digital forensics can sometimes link pseudonyms with other identities, especially as it is easy to peruse all of a user’s posts. Additionally, users who maintain multiple accounts can mistakenly post a message with the wrong account. Even though such users can edit or delete mistaken messages, it’s likely the original will survive elsewhere.

V1 was more cognizant of these concerns and stated they only used posts wherein Redditors explicitly declared they were using a throwaway (single-use) account. However, oddly, V1 did include verbatim quotes from a few Redditors who wrote *why they chose not to use a throwaway*. A researcher might inadvertently collect posts with the term “throwaway” even if the Redditor was explaining why they did *not* do so.

The research reports of V1 and V2 each had about twenty phrases (of ten or more words), and I was able to locate almost all of them using three indexes of Reddit content.

Reddit: Reddit provides native searching of all posts, via the website’s search bar and the Application Programming Interface (API). The search fields *author*, *title*, *selftext*, and *subreddit* can be useful in locating sources (Reddit Search, 2021). In 2017 Reddit dropped time-delimited searches (e.g., find results from between May – June 2019). Its ability to locate disguised messages is poor: it could succeed on eliminated words but failed on altered words and punctuation. (See the section “Limitations” for discussion of changes to the service.) The URL corresponding to a typical search is <https://www.reddit.com/r/{subreddit}/search/?q={source_phrase}&include_over_18=on.>
Google: Google indexes all of Reddit, which is especially useful for finding comments. Its searches can be narrowed by way of the time and site fields, though its time facet is often inaccurate. Google can locate disguised phrases. The URL corresponding to a typical search is <https://www.google.com/search?q=site:reddit.com r/{subreddit} {source_phrase}>.
RedditSearch (using Pushshift.io): Pushshift is a third-party copy of Reddit. It indexes posts and comments and provides many search fields via its API, including date and subreddit (Baumgartner et al., 2020; Baumgartner, 2016). Pushshift’s index can be a dozen or so hours out of date from Reddit and may keep data that has been edited or deleted on Reddit. It can also be incomplete (Gaffney & Matias, 2018). Though Pushshift provides an API, human-friendly webpage interfaces are provided by others, including RedditSearch.io. Often, Pushshift retains deleted posts, which can be searched for on human-friendly websites including Removeddit.com, Ceddit.com, and ReSavr.com. Pushshift can find disguised phrases. The URL corresponding to a typical search is <https://redditsearch.io/?term={source_phrase}&subreddits={subreddit}&searchtype=posts,comments&search=true&start=0&end=1611258724>

Table 2 represents the relative usefulness of the three search services across all 22 research reports. Oddly, Google under-performed (“†”) in verbatim searches because it did not return any of V1’s 18 sources from Reddit. Google’s search algorithms are opaque and ever-changing, so I do not know why it missed these posts, but they could become locatable in the future. Indeed, much could change, and search engines’ capabilities are likely to improve. When removing V1 from the calculation, Google’s verbatim rate is 45%.

**Table 2:** Percent of sources found (non-exclusively) at service

	Google	Reddit	RedditSearch
Verbatim	13% (45%†)	36%	52%
Reworded	32%	11%	52%

RedditSearch (using the Pushshift service) was the most generative search engine because it permits accurate time and subreddit searches. In practice, winnowing away misses is as important as roughly matching hits. It also returned some posts that had since been deleted by their authors, including from V1’s users who did *not* use throwaways – and perhaps regretted that decision and deleted their posts. Similarly, I was able to locate phrases from deleted posts in the reports of V1, R6, R14, and R18.

The deletion of messages by Redditors suggests that users can feel exposed even when using pseudonymous or throwaway accounts. Users should appreciate that deleted messages on Reddit can be archived and indexed off-site. Researchers should appreciate that they could inadvertently publicize such messages.

Additionally, the Pushshift data originally contained public *and private* subreddits (determined by moderators) and can include quarantined subreddits (determined by Reddit for problematic but not yet banned) (Stuck_In_the_Matrix, 2019, 2015). Pushshift data has also been packaged in common “big data” frameworks, permitting even more powerful queries and analysis. For example, BigQuery (Balamuta, 2018) was used by R5, R6, and R17; ConvoKit (2018) was used by R9. Locating sources via these resources would add additional capabilities beyond the human-facing searching engines I limited myself to.

### Making mistakes and the need for a system

V3 argued that because the site is premised on Redditors competing for upvoted visibility, the site can be taken as public. Even so, V3 elided all usernames, except for two central characters in their report. They quoted phrases from a couple of posts and a handful of comments. This made it easy to find their sources. I was also able to (redundantly) find a post by looking for V3’s description of a meme via a Google image search.

Upon reading V3’s report I was confused by the positioning of Redditors as authors deserving credit in a public venue (hence no consent was obtained), yet, also of a need to elide most Redditors’ usernames (while quoting their prose verbatim). V3 responded that the approach to sources and its description changed during the reports’ review and editing: “originally each of the pseudonyms was formally cited, but this was removed in an earlier stage of peer review.” The confusion in the description was the likely result of this change “and not picked up during the copy-editing stage of the journal.”

R12 also reflected on the likely cause of mistakenly including verbatim phrases. Because of the massive size of their data, “we only paraphrase those we would actually use in the paper.” The process of managing the manuscript and sources then became a problem: “We initially inserted the original quotes into the draft and did one round of paraphrasing. But writing was an iterative process, especially when review & resubmit was involved, during which we might switch in and out quotes as appropriate.” Having multiple authors work on this no doubt contributed: “We probably thought one person on the research team did the paraphrasing.”

Similarly, R16 intended to change all the quotes and believed they had: “I obviously didn’t do a thorough job at it, and I don’t know why – was I aiming to keep the authenticity of the quotes, or was I simply running out of time and did not work diligently? Probably both.” Ethical disguise had been at the forefront of their mind at the start, but perhaps not later: “Was I weighing up the risk of [sources] being identified in this context of technologies used by parents? Certainly in my ethics application, but probably not as much in the reporting.” R5, R6, R10, and R19 similarly included verbatim phrases contrary to their stated policy, perhaps because of similar reasons as the researchers above.

### Balancing Fidelity and disguise

Many of the interviewees spoke of the challenge of balancing fidelity to sources’ sentiments with the ethical concern of limiting sources’ exposure.

With respect to identities, V3 shared that “The intention here was to not explicitly name Redditors (using their pseudonym) unless there was a reason to do so.” That is, “My ethical practice defaulted to anonymity, but when necessary for the discussion I used the pseudonyms that the user provides to the public forum.” Two prominent Redditors “are named because of how identifiable their content is and how widely it has been shared across platforms (including sites such as KnowYourMeme).” Additionally, one “username itself was worthy of comment as a signifier of geek humor.” And, once published, the “study gave them significant appreciated notoriety on Reddit and beyond,” something they welcomed.

With respect to verbatim phrases, V3 recognized that phrases can be searched for. However, “What you can find this way is a user’s publicly available (shared) content and pseudonym, not their ‘real name’.” In any case, “As researchers we understand that ethics is a process, not something that is achieved once it is rubberstamped by an institution.” As part of V3’s process, “I considered the trade-off between potential tracking back to a pseudonym and fair representation. The expectation of users, popularity of content, and lack of real names also fed into this calculation.”

R2 attempted to disguise sources and this was a shift in practice from earlier work, where they “included the usernames and preserved quotations.” The earlier work had been influenced by an AoIR presentation about a site wherein the Redditors saw themselves as creative developers worthy of and preferring attribution. “And, because I believed part of my argument about Reddit hinged on the creative play that Redditors engaged in, I wanted to preserve usernames (as this is one of the places where this kind of play occurred).” However, “given the nature of the online sphere these days (this was pre-Gamergate), I would likely not have made the same choice.”

Additionally, the “AOIR guidelines have been hugely influential” in R2’s practice. The guidelines respect that research practices “are not one-and-done decisions, but that things like anonymizing [online] identities/quotes are ongoing decisions that we need to make. IRB guidelines are pretty much worthless in this regard, as they would consider any public forum ‘public’ and their understanding of how easy it is to find out information based on usernames or quotes is limited in my experience.”

The AoIR guidelines were influential to R3, R16, and R18 as well. Other noted influences included boyd (2007)﻿, Kozinets (2015), and especially Markham (2012).

### Changing practice and changing context

Unlike R2’s past work, in their present report, usernames were elided and phrases from posts and comments were lightly reworded. Though Google can be astoundingly good at finding similar phrases when the field is sufficiently narrow, the modest rewording was sufficient to frustrate my efforts with Reddit, Google, and RedditSearch. However, those messages appeared in threads whose titles were included verbatim in the report, and this leaked information was useful in locating sources. Once in the right thread, it was trivial to locate phrases from the report. Not only did verbatim titles become avenues for locating messages, but they can also be sensitive disclosures.

Like V3, R2 changed their level of disguise during the report’s review: “This piece was a content analysis, and so in my first draft of the article I actually preserved this material as-is, because I wanted to be transparent and make my study potentially replicable.” However, reviewers found this to be problematic because it could open the Redditors to trolling. R2’s forums were *not* sensitive *per se*: “I would absolutely have issues with someone using usernames/direct quotes from a health or relationship subreddit for obvious reasons.” Yet, personal disclosures were made in the studied forums and its users are sometimes targets of harassment. R2 agreed with the concern and altered the quoted phrases: “the outlet and the reviewers made a difference in this case.”

R2’s experiences speak to the importance of site-specific context and the larger zeitgeist. A practice on one subreddit might not be appropriate to another, especially after larger events increase the likelihood of trolling and harassment. Similarly, R15 noted that “I think ethical use of social media posts for research has to take context into consideration – it’s a different thing to quote someone posting from a public Twitter account than it is to burrow into an obscure subreddit and identify one comment on a long thread to surface.” And the social media context is dynamic: “With more and more news-style platforms grabbing social media posts without permission to use as comments in news articles we might even see a shifting culture around what people think is permissible once they’ve posted something publicly. Or this practice might result in pushback in which people demand to be asked permission or credited for their posts!”

The world that researchers seek to understand is ever-changing, as are the technical affordances of media platforms and search services. It can be difficult to match ethical policy to the quickly shifting online world, as can implementing that policy with consistent practice, especially given the time and changes needed during a reports’ publication.

### Effective tactics of disguise

R1’s report is a detailed ethnography of a few identified subreddits that is well-grounded with descriptions of community concerns and quotes from Redditors. Yet there were only two phrases (of more than 10 words) to attempt to locate. Most of everything else was from subreddits’ documentation (not sensitive) and interviews (not indexed by search engines). Confidential interviews of public Redditors can enable a surprising degree of richness, disclosure, *and* confidentiality.

Otherwise, searching for 150 + sources in the 22 research papers reveals that the metaphor of finding a needle in a haystack (of returned search results) is apt. Reports that focus on a single subreddit (as stated or inferred) in a single year winnow away much of the hay. Additionally, changes of punctuation, switching to or from contractions, single word insertions or removals, and retaining novel words are usually insufficient disguise.

Larger datasets – or less specific descriptions – and more substantive changes are more effective. R9, for example, included 11 effectively disguised phrases. Their dataset included over a million posts over a dozen subreddits – their haystack was large. Additionally, their technique for disguising the phrases might be an effective consequence of an analytic technique used to normalize text into a canonical form: “normalization is an approach to finding long English phrases in online vernacular and replace them with concepts in clinical knowledge sources. For instance, ‘shortness of breath’ in a post is replaced by the term ‘edema’ or ‘dyspnea.’”. Though this technique was not created to disguise sources, it seemingly serves that purpose.

### The rigor of testing and disciplinary differences

R3 used about ten reworded phrases from Reddit; I was not able to locate their sources.

Two influences at the start of R3’s career – as well as the sensitive topics they tend to study – led to a rigorous process for disguising sources. Today, their process is an iterative one, of swapping in synonyms or larger rephrasing “in a way that doesn’t change the meaning and yet would be untraceable. If someone were to put that quote in Google and try to find it there … they wouldn’t be able to do that.” To accomplish this, R3 performs the task themselves. That is, they seek to locate their own disguised sources – though, as seen above, Google is not the only index of Reddit messages. And their method is akin to my method here: using exact searches (in quote marks), near searches (without quote marks), and focusing on portions of a phrase, while conceding the process is “pretty subjective.” Just as with my method, they have to choose how to specify the search and how many returned results to review.

R3’s germinal influences were an event and a scholar. First, as a doctoral student, they saw another researcher’s source publicly disclosed because of the inclusion of a verbatim quotation. This was “an example of how [verbatim quotes] can be a problem.” Second, subsequently, R3 learned of Markham (2012)’s coining and explication of “ethical fabrication,” giving R3 a name and rationale for something similar to what R3 was already doing.

Today, in their role as an editor and reviewer, R3 sometimes asks researchers to reflect on their sourcing practice and rationale, with those in their discipline tending to be thoughtful about this issue. Elsewhere, though, R3 has experienced pushback against fabrication, such as in a presentation before a group of network-analytic sociologists. The audience was upset when they learned they were seeing fabricated, rather than authentic, quotes and images in the presentation.

R4 employed similar tactics as R3, changing “gender, location, specific details of an incident etc. so that, while they convey the original thought of the author, they cannot be traced back to them.” They tested these disguised phrases using a Pushshift-related service and “shared the two snippets with others in the team in order to see if the rephrase is too far off.”

R14 was the third interviewee to test their disguises. Though R14 used Pushshift in other work, they did not test their disguises against RedditSearch/Pushshift. Instead, they pursued the tactic of change-and-test “until I couldn’t find the quote + reddit in Google.” I was able to locate many of their sources because I limited my queries to the Reddit website (i.e., site:reddit.com) in Google. This extra specificity plus the year led to many of R14’s sources.

R16, too, has tested their disguises in the past via Google, “but I don’t know if I did it with this paper. And I certainly did not try other search engines.”

If a researcher wants to use disguised public data, rather than interviews, then the best disguise is tested disguise. This means investigating where their data sources are likely to be archived, how it all can be searched, and using as many facets of search as possible to test their efforts.

## Limitations and Future Work

The present work is idiosyncratic and relatively small in scale; nonetheless, it shows that the practice of disguise is often haphazard and ineffective. The next step is to investigate automated methods for managing and disguising sources. That is, can automated programs and services alter phrases for inclusion in reports with more or less efficacy than humans? Managing sources could be easy as keeping quotes, their source, and their disguise in a spreadsheet shared among collaborators – and this could then facilitate automatic testing. The next phase of the current work tests the feasibility and efficacy of this approach (Reagle & Gaur, 2022) .

The web and the services that index it are dynamic. Google routinely updates its search algorithms, and in April of 2022 – after the data and collection in the present report – Reddit announced they had extended their search facility to comments and made their searches less literal: “100% of a query doesn’t have to match the text of a post to return relevant results. By doing this we saw a 60% increase in results for queries that previously didn’t receive results” (Staff, 2022). Such changes will affect how easy it is to locate a source. Though such changes could make location more difficult by crowding out the true source, it is clearly the intention of services to improve their search efficacy.

Another practical follow-up is to increase the understanding of risks and options among researchers. King (1996) faulted Finn & Lavitt (1994) for leaking information about source context decades ago, and yet it still happens. A guide that builds on Bruckman (2002)’s categories of disguise and identifies risks and options available to the researcher could help authors, reviewers, and editors. The guide could include a checklist of things to attend to for a given category of risk and disguise. And it could be complemented by site-specific information about conventions and norms, and affordances around user identity and message persistence and locatability.

Finally, messages appeared in research reports that were subsequently deleted by their authors – even if from pseudonymous and throw-away accounts. This merits more attention and work-in-progress indicates throwaway accounts regularly – routinely even – delete posts in advice subreddits.

## Conclusions

There is no single research policy appropriate to disguising online sources. For example, community members might expect or appreciate recognition as creative authors. Like V3’s two Redditors who gained additional notice for appearing in her report, R18 noted that “One of the moderators of a subreddit I used reached out to me on ResearchGate and thanked me for my research and steps toward harm reduction; they were happy that I used material from their subreddit.”

If researchers chose to use disguise, however, their practice ought to effectively match their policy. I found descriptions of ethical policy that were confusing or inconsistent with actual practice. In a few cases, this was the result of changes made during the review and editing process. In another case, I suspect it was an oversight in data collecting and reporting. Many others simply made mistakes or failed to appreciate the affordances of Reddit and web searching.

The RedditSearch interface to the Pushshift repository proved especially useful in locating sources. And such data can be repackaged in ways that permit even more powerful searching capabilities (e.g., BigQuery and ConvoKit). While some researchers might use these resources in large-scale analyses, other researchers were unfamiliar with them. In addition to advanced search capabilities, these resources also mean that researchers who use them might include data since deleted by users in research analyses and reports.

The highest level of disguise, eliding usernames and rewording prose, can be effective, especially when the reworded phrases are tested against search engines – the practice of a few interviewed researchers. However, concerned researchers should be as specific as possible in their test queries, taking advantage of site, date, and subreddit facets.

My interviewees shared how their practices changed relative to their research sites, the larger cultural context, and their influences and experiences. The different approaches we see in reports, however, are not necessarily the result of a consistent policy (i.e., from conception to publication), fully cognizant of technical affordances (e.g., Google’s site: facet and RedditSearch/Pushshift existence and abilities), and users’ wishes (e.g., when users delete posts from throw-away accounts). The research community can improve on this, though, via similar site-specific investigations and practical guidelines that inform the conception, execution, and review of research. We also need additional work on automating, managing, and testing research disguise.

## Data Availability

Not applicable. Research data are: (1) research reports, (2) phrases from those reports taken from Reddit, and (3) interviews with the authors of the reports. The first two datasets are confidential so as not to embarrass researchers. The third set is confidential because they were obtained via a consent form that sated: “The confidentiality of your research records will be strictly maintained. Records of our discussion will be (1) kept separate from this consent form, (2) not shared with others, and (3) kept on an encrypted file system.”
